# Prophylactic epidural blood patch for cerebrospinal fluid leakage after intrathecal drug delivery system implantation in patients with refractory cancer pain: a multi-center retrospective cohort study

**DOI:** 10.3389/fneur.2026.1883156

**Published:** 2026-07-16

**Authors:** Aimin Zhang, Ying Chen, Qiju Li, Zikun Ning, Huaiming Wang, Hui Pan, Xin Min, Yao Wang, Deshan Li, Pengjiu Feng, Qin Li

**Affiliations:** 1 Department of Anesthesiology and Pain Medicine, Sichuan Clinical Research Center for Cancer, Sichuan Cancer Hospital & Institute, Sichuan Cancer Center, University of Electronic Science and Technology of China, Chengdu, China; 2 Department of Gynecologic Oncology, Sichuan Women's and Children's Hospital/Women's and Children's Hospital, Chengdu Medical College, Chengdu, China; 3 Department of Oncology, Chengdu Xinhua Hospital, Xinhua Hospital Affiliated to North Sichuan Medical College, Chengdu, China; 4 Graduate School, Guangxi University of Chinese Medicine, Nanning, China; 5 Department of Anesthesiology and Pain, The People's Hospital of Leshan, Leshan, China; 6 Department of Anesthesiology, Yanjiang District People's Hospital, Ziyang, China; 7 Department of Interventional Therapy, Sichuan Clinical Research Center for Cancer, Sichuan Cancer Hospital & Institute, Sichuan Cancer Center, University of Electronic Science and Technology of China, Chengdu, China; 8 Department of Pain Medicine, The Third Affiliated Hospital of Guangxi University of Chinese Medicine, Liuzhou Traditional Chinese Medical Hospital, Liuzhou, China

**Keywords:** cerebrospinal fluid leakage, intrathecal drug delivery system, propensity score matching, prophylactic epidural blood patch, refractory cancer pain

## Abstract

**Introduction:**

Current guidelines for postdural puncture cerebrospinal fluid (CSF) leakage advocate conservative management, extrapolated from general populations. Patients with refractory cancer pain (RCP) often have advanced disease, malnutrition, and hypoalbuminemia, which may compromise tissue repair and reduce the reliability of spontaneous dural closure. The role of prophylactic epidural blood patch (PEBP) in this high-risk subgroup is undefined.

**Methods:**

This multicenter retrospective cohort study enrolled RCP patients undergoing intrathecal drug delivery system (IDDS) implantation (Jan 2023–Aug 2025). Patients received either intraoperative PEBP (10–15 mL autologous blood) or conservative management (strict bed rest for 24–48 h, intravenous hydration 1,500–2,000 mL/day, oral caffeine). Propensity score matching (1:1) balanced recorded baseline and available procedural covariates. The primary outcome was moderate-to-severe postural headache syndrome (PHS).

**Results:**

After matching (78 patient pairs, *N* = 156), PEBP was associated with lower rates of moderate-to-severe PHS (6.41% vs. 25.64%; *p* < 0.001), exploratory imaging-confirmed CSF leakage (7.69% vs. 24.36%; *p* = 0.003), and remedial EBP requirement (2.56% vs. 15.38%; *p* = 0.006). The absolute risk reduction for moderate-to-severe PHS was 19.23%, corresponding to a number needed to treat (NNT) of approximately 5. Headache duration and hospital stay were also shorter in the PEBP group. Subgroup findings were exploratory. Minor adverse events occurred in 11.54% of PEBP patients. No clinically evident neuraxial seeding was observed during short-term follow-up (median 3 months, range 2–5 months).

**Conclusion:**

In this retrospective cohort, intraoperative PEBP was associated with fewer early CSF leakage-related complications and shorter recovery after adjustment for recorded covariates. These findings support further prospective evaluation of PEBP in carefully selected patients. Prospective randomized controlled trials are warranted to validate these results. Causal inference, long-term safety, and patient-centered benefit require confirmation.

## Highlights

- Why was this study conducted?—Intrathecal drug delivery systems (IDDS) are an established option for carefully selected patients with refractory cancer pain (RCP), but dural puncture during implantation increases cerebrospinal fluid (CSF) leakage risk. In patients with advanced cancer, malnutrition and hypoalbuminemia may reduce the reliability of spontaneous dural closure. The safety and efficacy of prophylactic epidural blood patch (PEBP) in this high-risk cohort remained unproven.
- What were the key findings?—In this propensity score-matched multicenter study (78 patient pairs), PEBP was associated with lower rates of moderate-to-severe postural headache syndrome, exploratory imaging-confirmed CSF leakage, and remedial EBP need. Headache duration and hospital stay were shorter in the PEBP group; subgroup findings were exploratory. Only minor, self-limiting adverse events (11.54%) were observed, and no clinically evident neuraxial seeding was detected during short-term follow-up.
- What are the clinical implications?—PEBP may reduce early CSF leakage-related complications by providing an immediate mechanical seal at the dural defect. These data support prospective evaluation of PEBP in carefully selected patients, rather than establishing definitive clinical superiority. Prospective randomized trials are warranted to validate findings.

## 1 Introduction

Refractory cancer pain (RCP), characterized by inadequate pain control despite optimized opioid therapy or the presence of intolerable treatment-related adverse effects, affects approximately 10%–20% of patients with advanced malignancies, profoundly diminishing quality of life and complicating clinical management (1, 2). Intrathecal drug delivery systems (IDDS) are an established interventional option for carefully selected patients with RCP, particularly when conventional systemic analgesic strategies provide insufficient relief or cause intolerable adverse effects (3, 4). However, the requisite dural puncture during IDDS implantation introduces a risk of cerebrospinal fluid (CSF) leakage (5), a complication that may be exacerbated in the RCP population due to underlying pathophysiological vulnerabilities (6). The primary goal of IDDS in this context is to improve the quality of the patient’s remaining life by providing superior analgesia, even when overall survival may be limited.

CSF leakage-induced postural headache syndrome (PHS), manifesting as orthostatic headache, neck stiffness, and visual disturbances, significantly augments patient suffering, prolongs recovery, and escalates healthcare utilization (7). Contemporary international guidelines, primarily informed by evidence from obstetric and general surgical cohorts, advocate conservative management—encompassing bed rest, hydration, and caffeine supplementation—over prophylactic interventions for postdural puncture CSF leakage (8). This recommendation is predicated on the observation that dural defects in otherwise healthy individuals typically heal spontaneously, facilitated by robust nutritional status and minimal systemic comorbidities (9). Patients with RCP, however, present a distinct clinical profile that may limit the applicability of these guidelines.

Advanced cancer is commonly associated with malnutrition, cachexia, systemic inflammation, and hypoalbuminemia, which are linked to impaired tissue repair and poorer postoperative recovery (10). Therefore, conservative strategies that rely mainly on spontaneous dural closure may be less reliable in selected patients with RCP. While epidural blood patch (EBP) is the therapeutic standard for refractory PHS, its prophylactic use epidural blood patch (PEBP) in cancer patients is contentious, primarily due to theoretical concerns about neuraxial malignant seeding (11).

Alternative prophylactic materials such as fibrin glue and hydroxyethyl starch (HES) have been investigated in non-cancer populations (12–14). Consequently, the potential role of PEBP in preventing cerebrospinal fluid leakage-related complications specifically in patients with refractory cancer pain undergoing intrathecal drug delivery system implantation remains largely unexplored. This gap in knowledge directly impacts clinical decision-making and warrants further investigation. To address this critical knowledge gap, we performed a propensity score-matched retrospective cohort study assessing the efficacy and safety of intraoperative PEBP in patients with RCP undergoing IDDS implantation.

## 2 Methods

### 2.1 Study design and population

This was a non-randomized, observational, retrospective cohort study conducted across six tertiary medical centers in China:

1. Sichuan Cancer Hospital & Institute, Chengdu.
2. Sichuan Gem Flower Hospital, Chengdu.
3. Chengdu Xinhua Hospital, Chengdu.
4. The Third Affiliated Hospital of Guangxi University of Chinese Medicine, Liuzhou.
5. The People’s Hospital of Leshan, Leshan.
6. Yanjiang District People’s Hospital, Ziyang.

Consecutive cases of patients with RCP who underwent semi-implantable IDDS implantation during the case observation period from January 2023 to August 2025 were retrospectively screened and included; this interval refers to the clinical diagnosis/treatment period of the included cases, not the date of ethics approval, data extraction, or statistical analysis. All implantation procedures adhered to a unified institutional protocol (3), which standardized the operating room environment, skin preparation with chlorhexidine-alcohol, needle insertion technique (paramedian or midline approach based on anatomical landmarks), and catheter fixation method. The decision to perform PEBP was based on institutional protocol and surgeon preference rather than on specific intraoperative risk factors. While this introduces potential for selection bias, it reflects real-world practice. Detailed intraoperative data on the number of dural puncture attempts, needle gauge, operator experience, and visible CSF leak were not systematically recorded; this is the central methodological vulnerability of this retrospective analysis. Consequently, treatment estimates should be interpreted as adjusted associations rather than causal effects.

Inclusion criteria were as follows: (1) Pathologically confirmed malignant tumor with RCP, defined as persistent pain (Numerical Rating Scale [NRS] ≥ 4) and/or breakthrough pain (≥ 3 episodes/day) following 1–2 weeks of standardized opioid therapy, or intolerable opioid-related adverse effects (15); (2) Underwent Semi-implantable IDDS implantation between January 2023 and August 2025; (3) Availability of complete clinical and follow-up data.

Exclusion criteria comprised: (1) Active systemic or localized surgical site infection; (2) Significant coagulation disorders (platelet count < 80 × 10^9^/L, prothrombin time prolongation >3 s, activated partial thromboplastin time prolongation >10 s) (16); (3) Documented allergy to implanted device components or intrathecal medications; (4) Spinal canal metastasis or other conditions potentially disrupting normal CSF circulation (16); (5) Administration of a therapeutic EBP following PHS onset but prior to the assessment of study outcomes, as this would confound the natural history and the evaluation of the prophylactic strategy; (6) Incomplete medical records or loss to follow-up.

### 2.2 Ethical approval

This study received primary ethical approval from the Ethics Committee of Sichuan Cancer Hospital (Ethical Approval Number: KY-2026-054-02, Supplementary file). The clinical period from January 2023 to August 2025 denotes the diagnosis/treatment period of the included cases; retrospective data extraction and analysis were conducted after ethical approval was obtained, and therefore the 2026 approval number is consistent with the retrospective study design. All six participating tertiary medical centers fully acknowledged and accepted this primary ethical approval, and additionally completed local ethical review and obtained formal approval in accordance with their respective institutional regulations. The local ethical approval numbers for each participating center are available upon request from the corresponding author. All procedures involving human participants, including the PEBP intervention, were conducted in accordance with the ethical principles set forth in the Declaration of Helsinki. Because the present study analyzed previously recorded, de-identified clinical data, the requirement for individual informed consent for the retrospective data analysis was waived by the ethics committee; documentation of the waiver is available from the corresponding author upon reasonable request. Written consent for the clinical IDDS implantation and, when applicable, PEBP procedures was obtained according to routine institutional practice before treatment.

### 2.3 Intervention protocols

Following dural puncture, patients were managed according to one of two intraoperative preventive strategies:

(1) *PEBP group:* Following catheter placement and before surgical closure, using strict aseptic technique, 10–15 mL of autologous venous blood was slowly injected into the epidural space at the adjacent vertebral level at a rate of approximately 1–2 mL/s. The absence of intrathecal placement was confirmed by intermittent aspiration for CSF. Patients remained supine for 24 h postoperatively.
(2) *Control group:* Patients received strict bed rest (head of bed elevation ≤ 15 degrees) for 24–48 h, intravenous crystalloid hydration (1,500–2,000 mL of normal saline or balanced crystalloid per day), oral caffeine citrate (200 mg three times daily), and analgesia as needed. While these protocols were prescribed, adherence to bed rest duration and actual fluid intake could not be objectively verified in this retrospective study.

### 2.4 Outcome measures

*Primary outcome:* Incidence of postoperative moderate-to-severe PHS. PHS was diagnosed according to International Association for the Study of Pain criteria, with severity graded as (17): Mild (orthostatic headache relieved by recumbency, no accompanying symptoms, no additional analgesia); Moderate (orthostatic headache not fully relieved by recumbency, accompanied by 1–2 symptoms [e.g., neck stiffness, tinnitus], requiring oral analgesia); Severe (persistent headache even when supine, accompanied by ≥ 3 symptoms [e.g., visual disturbance, vertigo, nausea/vomiting], requiring intravenous analgesia or invasive intervention). Severity was graded by the treating clinicians, and final classifications were verified by two independent researchers blinded to group allocation to ensure consistency.

*Secondary outcomes included:* (1) Exploratory imaging-confirmed CSF leakage, defined as MRI myelography-confirmed extradural fluid collection contiguous with the operative dural puncture site among patients imaged because of clinical suspicion, with images centrally adjudicated by two blinded radiologists across all centers; (2) Duration of PHS (time from onset to complete symptom resolution sustained for 48 consecutive hours); (3) Rate of remedial EBP administration; (4) Postoperative pain intensity (NRS scores at 1 week); (5) Hospital length of stay (LOS); (6) 30-day readmission rate attributable to CSF leakage-related complications; (7) PEBP-related adverse events, categorized as local (back pain, puncture site hematoma) or systemic (infection, nerve injury, suspected malignant cell seeding, fever).

### 2.5 Data collection

After ethical approval was obtained, data were retrospectively extracted from the electronic medical record system and included: (1) Demographic characteristics (age, sex, body mass index [BMI], preoperative albumin); (2) Tumor-specific variables (histological type, TNM stage, presence of distant metastases); (3) Surgical details (vertebral puncture level, total operative time, intraoperative blood loss); (4) Perioperative data (preoperative anticoagulant/antiplatelet use, postoperative analgesic requirements, PHS onset and duration, results of CSF leakage investigations, LOS, documented adverse events). Follow-up was conducted via outpatient clinical assessments and structured telephone interviews at 1 month and 3 months post-discharge to capture 30-day readmission rates and any delayed adverse events.

### 2.6 Statistical analysis

All statistical analyses were performed using SPSS version 26.0 (IBM Corp., Armonk, NY, USA) and R version 4.2.0 (R Foundation for Statistical Computing, Vienna, Austria) with the MatchIt, lmtest, and forestplot packages. Continuous variables were compared using independent t-tests or Mann–Whitney U tests as appropriate. Categorical variables were compared using χ^2^ or Fisher’s exact test.

**Figure 1:**
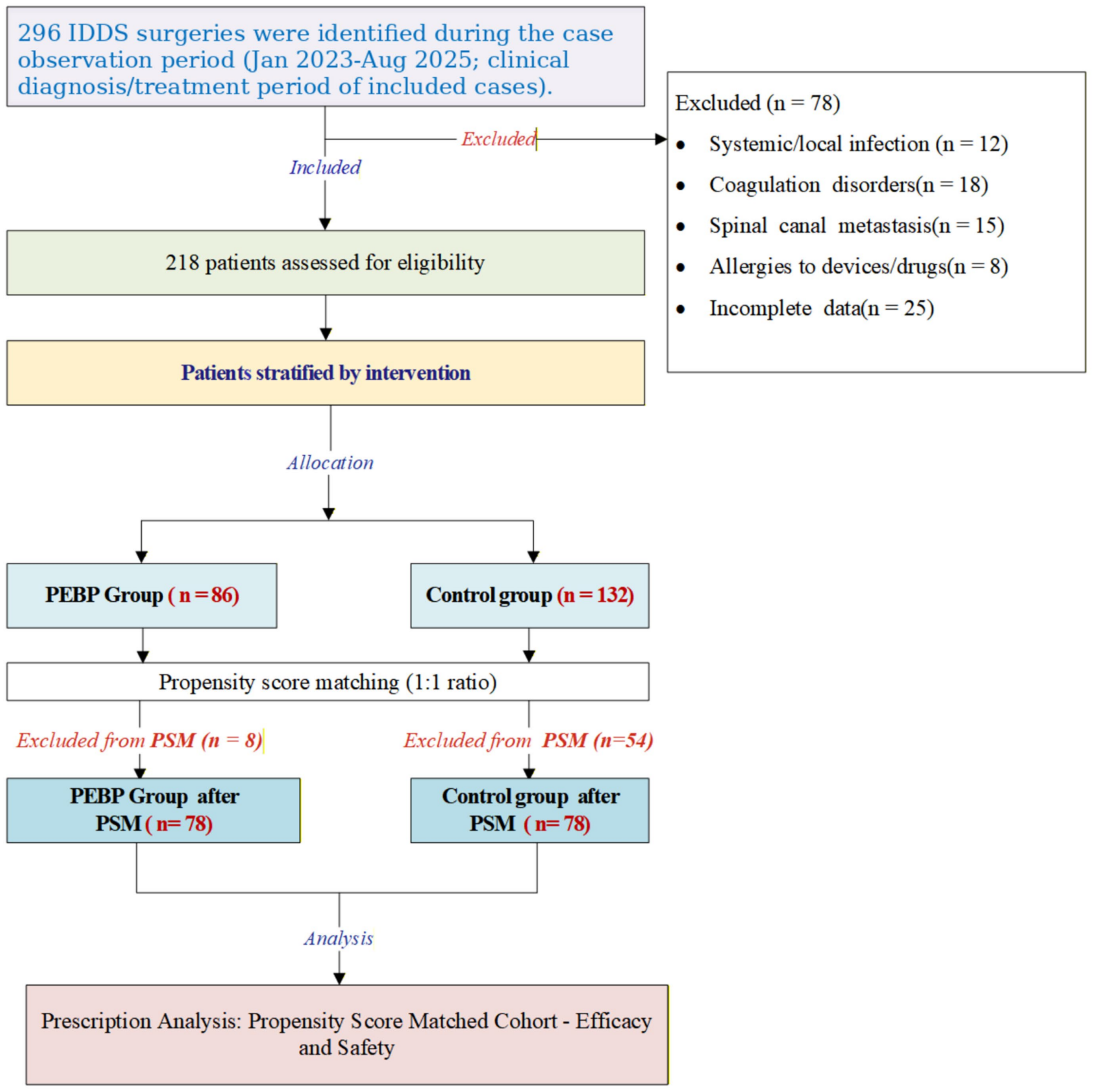
CONSORT-style flow diagram illustrating patient screening, eligibility assessment, and the propensity score matching process. The number of patients excluded due to non-matching is shown for each group.

To reduce imbalance in recorded covariates, propensity score matching (PSM) was performed; however, this approach could not address unmeasured intraoperative confounding. The propensity score (probability of receiving PEBP) was estimated via multivariable logistic regression. The full list of covariates entered into the model was: age, sex, BMI, preoperative albumin, tumor type, TNM stage, distant metastasis status, vertebral puncture level, operative time, intraoperative blood loss, and preoperative anticoagulant/antiplatelet use. A 1:1 nearest-neighbor matching algorithm without replacement with a caliper width of 0.02 of the logit of the propensity score was applied. There was no missing data for the covariates included in the PS model. The number of patients excluded due to non-matching is reported in Figure 1. The distribution of propensity scores before and after matching was examined to ensure overlap/positivity (Supplementary Figure S1). Covariate balance was assessed using standardized mean differences (SMD), with an SMD < 0.1 considered indicative of adequate balance (Supplementary Figure S1) (18). The primary matched estimand was restricted to patients within the region of common support. Patients outside the matching caliper, including unmatched controls, were excluded from the primary matched effect estimates; therefore, the estimates should be interpreted for the matched/common-support population rather than the full enrolled control cohort.

Treatment effects are reported as relative risk (RR) or mean difference (MD) with 95% confidence intervals (CI). Absolute risk reductions (ARR) with 95% CIs are also reported. The number needed to treat (NNT) was calculated for the primary outcome. To contextualize the possible influence of unmeasured confounding, we calculated E-values for the primary outcome. The E-value represents the minimum strength of association that an unmeasured confounder would need to have with both the treatment and the outcome to fully explain away the observed association. Time-to-event data for PHS-free survival were visualized using Kaplan–Meier curves and compared using the log-rank test. For the Kaplan–Meier analysis, the time origin was the time of IDDS implantation. Patients were censored at the time of loss to follow-up, death, or the end of the 30-day follow-up period. No competing events that would preclude the occurrence of PHS were identified. All statistical tests were two-sided, with *p* < 0.05 considered statistically significant. Because this was a fixed-time-window multicenter retrospective cohort rather than a prospective randomized trial, no formal *a priori* sample-size calculation was performed. E-values should be interpreted cautiously because they do not remove confounding and cannot compensate for the absence of key intraoperative variables in the dataset. Any presentation of outcomes among unmatched controls is intended only as a descriptive transparency supplement and was not combined with the matched-cohort estimates.

## 3 Results

### 3.1 Study population and PSM matching process

During the case observation period, 296 consecutive patients who received relevant diagnosis/treatment were retrospectively screened from the medical records. After applying inclusion/exclusion criteria, 218 cases were included (PEBP: *n* = 86; Control: *n* = 132). Following 1:1 PSM, 78 well-matched patient pairs (*N* = 156) were generated for final analysis (Figure 1). A total of 8 patients from the PEBP group and 54 from the control group were excluded from the matched analysis due to falling outside the caliper. The distribution of patients by center and year is presented in Supplementary Table S1, showing variability in PEBP adoption across centers but no significant temporal shift in allocation ratio during the observation period. Thus, the matched control group represented 78/132 (59.1%) of enrolled controls, while 54/132 (40.9%) controls were outside the matching common-support region. Accordingly, the primary matched comparison reflects a selected common-support population rather than the entire enrolled control cohort. No additional patients were lost to follow-up during matching; however, 62 patients were excluded from the matched analytic cohort because they fell outside the caliper, with exclusion disproportionately affecting the control group. The outcomes of unmatched controls are interpreted descriptively for transparency only and should not be mixed with the matched-cohort adjusted estimates.

### 3.2 Baseline characteristics before and after PSM

Of 296 screened patients, 218 were included and 78 excluded. Key demographic and clinical characteristics (age, gender, BMI, preoperative albumin, TNM stage) were well-balanced between groups (all *p* > 0.05, *SMD* < 0.1; Supplementary Table S2). Significant differences were restricted to predefined exclusion criteria: spinal metastasis (0% vs. 15.4%), coagulopathy (0% vs. 12.8%), active infection (0% vs. 10.3%), and incomplete data/loss to follow-up (0% vs. 61.5%; all *p* < 0.001). These discrepancies reflect appropriate application of exclusion criteria, not inadvertent selection bias.

Prior to matching, the PEBP (*n* = 86) and control (*n* = 132) groups differed in age, preoperative albumin, TNM stage IV proportion, operative time, and intraoperative blood loss (all *p* < 0.05, *SMD* > 0.38; Table 1). After 1:1 propensity score matching (78 pairs), all baseline confounders were effectively balanced (all *p* > 0.05, *SMD* < 0.1; Table 2), including all variables entered into the propensity score model, supporting adjusted comparisons of recorded covariates while not excluding residual confounding from unmeasured intraoperative factors.

**Table 1:** Baseline characteristics before propensity score matching.

Characteristics	Total (*n* = 218)	PEBP group (*n* = 86)	Control group (*n* = 132)	*P*-value	SMD
Age, years (mean ± SD)	60.1 ± 9.0	62.3 ± 8.5	58.6 ± 9.2	0.002*	0.41
Gender, *n* (%)				0.851	0.09
Male	125 (57.3)	49 (57.0)	76 (57.6)		
Female	93 (42.7)	37 (43.0)	56 (42.4)		
BMI, kg/m^2^ (mean ± SD)	21.8 ± 3.4	21.3 ± 3.2	22.1 ± 3.5	0.087	0.23
Preoperative albumin, g/L (mean ± SD)	34.2 ± 5.0	32.5 ± 4.8	35.2 ± 5.1	<0.001	0.55
TNM stage, *n* (%)				0.015	0.38
III	68 (31.2)	23 (26.7)	45 (34.1)		
IV	150 (68.8)	63 (73.3)	87 (65.9)		
Operation time, min (mean ± SD)	61.2 ± 15.0	65.2 ± 15.3	58.6 ± 14.8	0.001	0.44
Intraoperative blood loss, mL (mean ± SD)	24.7 ± 9.6	28.5 ± 10.2	22.3 ± 8.6	<0.001	0.65

^*^P < 0.05 for a between-group comparison before matching. BMI, body mass index; TNM, Tumor Node Metastasis; SD, standard deviation; PEBP, Prophylactic Epidural Blood Patch; SMD, Standardized Mean Difference (SMD < 0.1 indicates good balance).

**Table 2:** Baseline characteristics after propensity score matching (1:1).

Characteristics	PEBP group (*n* = 78)	Control group (*n* = 78)	*P*-value	SMD
Age, years (mean ± SD)	61.5 ± 8.2	60.8 ± 8.5	0.653	0.08
Gender, *n* (%)			0.764	0.05
Male	45 (57.7)	47 (60.3)		
Female	33 (42.3)	31 (39.7)		
BMI, kg/m^2^ (mean ± SD)	21.5 ± 3.1	21.8 ± 3.3	0.582	0.09
Preoperative albumin, g/L (mean ± SD)	33.1 ± 4.6	33.5 ± 4.8	0.615	0.08
Tumor type, *n* (%)			0.812	0.06
Lung	30 (38.5)	32 (41.0)		
GI	28 (35.9)	26 (33.3)		
Other	20 (25.6)	20 (25.6)		
TNM stage, *n* (%)			0.728	0.07
III	20 (25.6)	22 (28.2)		
IV	58 (74.4)	56 (71.8)		
Distant metastasis, *n* (%)	62 (79.5)	60 (76.9)	0.845	0.06
Vertebral puncture level, *n* (%)			0.691	0.08
L2-L3	45 (57.7)	42 (53.8)		
L3-L4	33 (42.3)	36 (46.2)		
Operation time, min (mean ± SD)	63.5 ± 14.6	62.8 ± 15.1	0.731	0.05
Intraoperative blood loss, mL (mean ± SD)	26.8 ± 9.5	25.9 ± 9.1	0.567	0.10
Pre-op anticoagulant/antiplatelet use, *n* (%)	12 (15.4)	10 (12.8)	0.821	0.07

^*^P < 0.05 for a between-group comparison before matching. BMI, body mass index; GI, Gastrointestinal tumors; TNM, Tumor Node Metastasis; SD, standard deviation; PEBP, Prophylactic Epidural Blood Patch; SMD, Standardized Mean Difference (SMD < 0.1 indicates good balance).

### 3.3 Primary and secondary outcomes

The comparative outcomes are summarized in Table 3. The PEBP group was associated with a lower observed incidence of moderate-to-severe PHS after matching (6.41% vs. 25.64%; RR 0.25, 95% CI, 0.10–0.63; *p* < 0.001). The absolute risk reduction was 19.23% (95% CI, 8.34% to 30.12%), corresponding to a NNT of 5 (95% CI, 4 to 12). The E-value for the point estimate of this primary outcome was 7.2, suggesting that an unmeasured confounder would need to be associated with both treatment and outcome by a risk ratio of 7.2 each to explain away the observed effect (Supplementary Table S3). Similarly, rates of exploratory imaging-confirmed CSF leakage (7.69% vs. 24.36%; RR 0.32, 95% CI, 0.14–0.73; *p* = 0.003) and need for remedial EBP (2.56% vs. 15.38%; RR 0.17, 95% CI, 0.04–0.71; *p* = 0.006) were lower in the PEBP group. Of the 30 patients with exploratory imaging-confirmed CSF leakage. MRI myelography was performed in 8/78 (10.3%) patients in the PEBP group and 22/78 (28.2%) patients in the control group when CSF leakage was clinically suspected, rather than according to a protocol-mandated screening schedule. Among patients with exploratory imaging-confirmed CSF leakage (6/78 in the PEBP group and 19/78 in the control group), positive MRI myelography findings were characterized by extradural contrast or fluid tracking contiguous with the operative dural puncture/catheter entry site, with or without localized epidural or paraspinal fluid collection. Therefore, exploratory imaging-confirmed CSF leakage should be interpreted as a clinically triggered exploratory imaging-confirmed outcome rather than a systematically ascertained secondary endpoint. Because MRI myelography was performed more frequently in the control group than in the PEBP group, this endpoint is vulnerable to verification/ascertainment bias and may systematically undercount imaging-confirmed leakage events in the PEBP group. The primary clinical outcome of the study remained moderate-to-severe PHS, not MRI detection rate. Patients receiving PEBP also experienced a shorter duration of headache (3.21 ± 1.56 vs. 6.89 ± 2.34 days; MD −3.68, 95% CI, −4.38 to −2.98; *p* < 0.001) and a reduced hospital LOS (8.52 ± 2.13 vs. 12.36 ± 3.45 days; MD −3.84, 95% CI, −4.74 to −2.94; *p* < 0.001). Given the potential for skewness, median (IQR) values for headache duration [PEBP: 3.0 (2.0–4.0) vs. Control: 7.0 (5.0–9.0) days] and LOS [PEBP: 8.0 (7.0–10.0) vs. Control: 12.0 (10.0–15.0) days] are also presented in Table 3. A post-hoc mixed-effects model with center as a random effect yielded a similar adjusted association for the primary outcome (adjusted OR 0.21, 95% CI, 0.07–0.58).

**Table 3:** Primary and exploratory secondary outcomes after propensity score matching.

Outcome measures	PEBP group (*n* = 78)	Control group (*n* = 78)	Statistic	*P*-value	Effect size (95% CI)
Moderate-to-severe PHS, *n* (%)	5 (6.41)	20 (25.64)	*χ^2^* = 11.72	< 0.001	RR 0.25 (0.10–0.63) ARR 19.23% (8.34–30.12%) NNT 5 (4–12)
Exploratory imaging-confirmed CSF leakage, *n* (%)	6 (7.69)	19 (24.36)	*χ^2^* = 8.76	0.003	RR 0.32 (0.14–0.73)
Remedial EBP, *n* (%)	2 (2.56)	12 (15.38)	*χ^2^* = 8.12	0.006	RR 0.17 (0.04–0.71)
Headache duration, days (mean ± SD) [Median (IQR)]	3.21 ± 1.56 [3.0 (2.0–4.0)]	6.89 ± 2.34 [7.0 (5.0–9.0)]	*t* = 9.54	< 0.001	MD −3.68 (−4.38 to −2.98)
LOS, days (mean ± SD) [Median (IQR)]	8.52 ± 2.13 [8.0 (7.0–10.0)]	12.36 ± 3.45 [12.0 (10.0–15.0)]	*t* = 8.98	< 0.001	MD −3.84 (−4.74 to −2.94)
Postoperative NRS (1 week)	3.1 ± 1.0	3.3 ± 1.1	*t* = 1.09	0.356	MD −0.20 (−0.50 to 0.10)
30-day readmission rate, *n* (%)	2 (2.56)	3 (3.85)	Fisher’s exact	0.684	RR 0.66 (0.11–3.97)

PHS, postural headache syndrome; CSF, cerebrospinal fluid; PEBP, Prophylactic Epidural Blood Patch; EBP, epidural blood patch; NRS, Numerical Rating Scale; RR, relative risk; MD, mean difference; CI, confidence interval; ARR, absolute risk reduction; NNT, Number needed to treat; IQR, Interquartile Range.

### 3.4 PHS-free survival analysis

**Figure 2:**
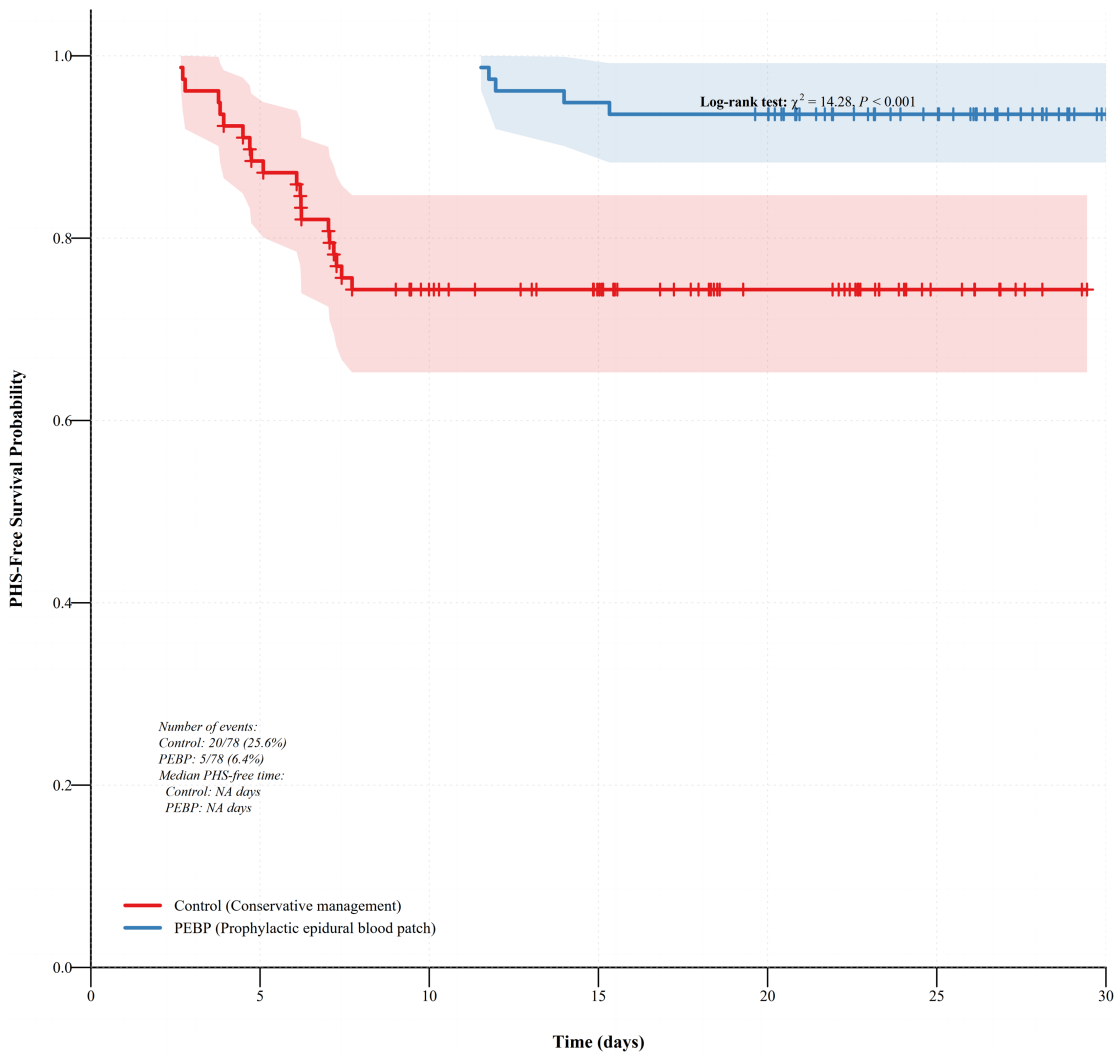
Kaplan–Meier curves illustrating PHS-free survival in the PEBP and control groups after propensity score matching. Time origin is the time of IDDS implantation. Patients were censored at loss to follow-up, death, or 30 days.

Kaplan–Meier analysis showed higher observed PHS-free survival in the PEBP group compared with the control group following propensity score matching (log-rank χ^2^ = 14.28, *p* < 0.001; Figure 2). The survival curves diverged early in the postoperative period. The cumulative incidence of PHS was lower in the PEBP group throughout the observation period, with the majority of events in the control group occurring within the first week. No crossover of the survival curves was observed.

### 3.5 Subgroup analysis of moderate-to-severe PHS

**Figure 3:**
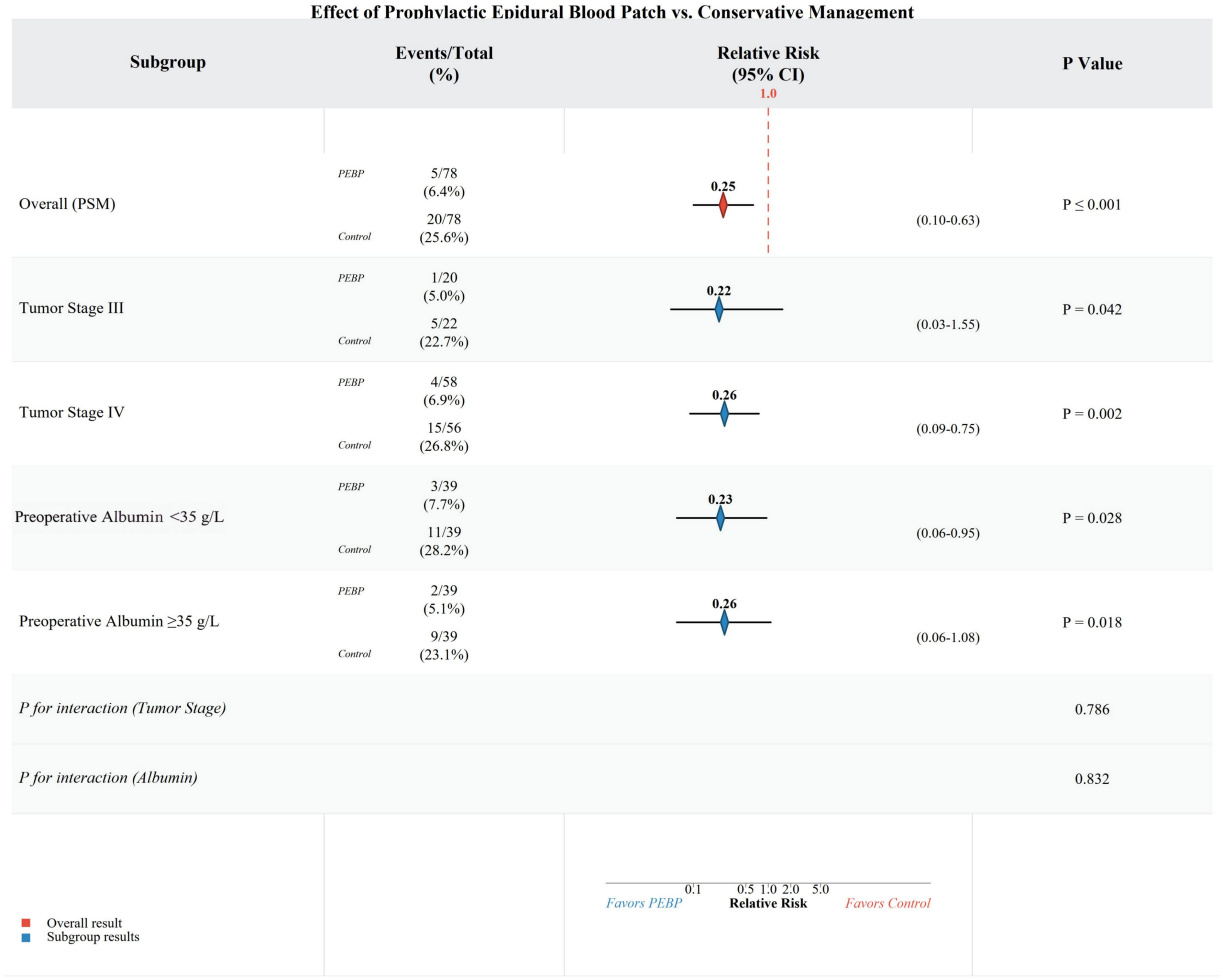
Forest plot summarizing subgroup analyses for the effect of PEBP on moderate-to-severe PHS incidence.

Exploratory subgroup analyses showed directionally similar associations across clinically relevant strata (Figure 3). Lower observed moderate-to-severe PHS incidence was seen in patients with both stage III (5.00% vs. 22.73%, *p* = 0.042) and stage IV tumors (6.90% vs. 26.79%, *p* = 0.002). Similarly, associations favored PEBP in both preoperative albumin strata, including < 35 g/L (7.69% *vs.* 28.20%, *p* = 0.028) and ≥ 35 g/L (5.12% *vs.* 23.07%, *p* = 0.018). Formal tests for interaction were non-significant for both tumor stage (P for interaction = 0.786) and albumin level (P for interaction = 0.832),. suggesting no clear evidence of effect modification, although these analyses were exploratory.

### 3.6 Safety profile

The safety analysis is detailed in Table 4. In the PEBP group, 9 patients (11.54%) experienced minor adverse events attributed to the procedure: transient puncture site back pain (*n* = 6, 7.69%) and low-grade fever (*n* = 3, 3.85%). All resolved spontaneously or with simple measures. No severe procedure-related adverse events such as infection, neurological injury, or symptomatic hematoma were recorded during the available follow-up. No clinically evident neuraxial seeding was observed during short-term follow-up (median 3 months, range 2–5 months); however, this short observation window cannot exclude rare or delayed neuraxial seeding events. In contrast, the control group had a higher overall incidence of adverse events related to CSF leakage/PHS or its management (20.51%), including gastrointestinal side effects from oral analgesics (*n* = 4), prolonged hypoalbuminemia (*n* = 6), and headache-related depressive symptoms (*n* = 6).

**Table 4:** Adverse events observed in the two groups after propensity score matching.

Adverse event	PEBP group (*n* = 78)	Control group (*n* = 78)	*P*-value
Any AE	9 (11.54)	16 (20.51)	0.136
PEBP-related AEs			
Back pain	6 (7.69)	0 (0.00)	< 0.001
Transient fever	3 (3.85)	0 (0.00)	0.022
CSF leakage/PHS-related AEs	0 (0.00)	16 (20.51)	< 0.001
Gastrointestinal reactions	0 (0.00)	4 (5.13)	0.047
Prolonged hypoalbuminemia*	0 (0.00)	6 (7.69)	0.012
Depression**	0 (0.00)	6 (7.69)	0.012
Severe AEs (including seeding)	0 (0.00)	0 (0.00)	–

AE, Adverse Event; PEBP, Prophylactic Epidural Blood Patch; CSF, Cerebrospinal Fluid; PHS, Postural Headache Syndrome. *Defined as a decrease in serum albumin of >5 g/L from baseline after 7 days of bed rest. **Defined as a new clinical diagnosis of depression or a PHQ-9 score ≥10 following the onset of PHS.

## 4 Discussion

This propensity score-matched multicenter study showed an adjusted association between intraoperative PEBP and lower rates of early CSF leakage-related complications in RCP patients undergoing IDDS implantation. The absolute risk reduction of 19.23% for moderate-to-severe PHS (NNT ≈ 5) suggests potential clinical relevance. Because IDDS remains a specialized intervention with uneven adoption across routine clinical settings, reducing peri-implantation CSF leakage-related morbidity may be particularly relevant to treatment acceptability and implementation.

A plausible explanation for the observed association is that PEBP reduces reliance on spontaneous dural closure in patients with advanced cancer, among whom malnutrition, cachexia, systemic inflammation, and hypoalbuminemia may compromise tissue repair (19). By providing an immediate mechanical tamponade, PEBP may seal the dural defect and restore CSF pressure while spontaneous healing occurs.

This study contributes unique observational data from a highly specialized population—patients with advanced cancer and refractory pain—among whom cachexia, hypoalbuminemia, and impaired nutritional status may be clinically relevant when evaluating dural-healing strategies (20). These findings thus provide a context-specific addition to the existing evidence base, meriting consideration in the ongoing evaluation of perioperative strategies for this complex patient group.

Our data reflects this vulnerability: even with standard conservative care, 25.64% of control patients developed moderate-to-severe PHS, a rate substantially higher than the 1%–5% typically reported in general populations (21). The epidural blood patch addresses this pathophysiological deficit primarily through immediate mechanical tamponade. The injected autologous blood forms a clot over the dural defect, providing a primary seal. Concurrently, by compressing the thecal sac, it facilitates a rapid restoration of CSF pressure, thereby alleviating the traction on pain-sensitive intracranial structures that underlies postural headache. This direct mechanical intervention counteracts the core issue of persistent CSF leakage in susceptible patients (22). This targeted approach directly counteracts the core pathophysiological limitation of impaired healing in RCP patients.

Our findings add context to the existing literature. While studies by Zhou et al. and Fan et al. support prophylactic strategies in non-cancer populations (12, 14, 23), evidence remains limited in oncological populations. In the context of RCP, autologous blood offers distinct theoretical advantages over synthetic materials: it eliminates risks of allergic reaction or foreign body response, and its content of platelets and growth factors (e.g., PDGF, TGF-beta) may promote more physiological repair. Crucially, the theoretical risk of malignant cell seeding, a concern for any epidural injection in cancer patients (24), is not augmented by introducing foreign substances. Autologous blood remains clinically attractive because it avoids foreign material and provides a direct mechanical seal; however, its long-term safety in advanced cancer requires further study. No clinically evident neuraxial seeding was observed during short-term follow-up, but this observation cannot exclude rare or delayed seeding events.

We observed no infection or neurological injury attributable to PEBP during the perioperative period, and no clinically evident neuraxial seeding was detected during short-term follow-up. The minor adverse events recorded (11.54%), consisting of transient back pain and low-grade fever, were self-limiting and manageable. Conversely, the conservative management strategy was associated with a distinct set of complications, including medication side effects, nutritional depletion from prolonged bed rest, and psychological morbidity from uncontrolled headache, highlighting that ‘conservative’ management is not without risk in this frail population (25). Low-grade fever occurred only after PEBP, resolved without sequelae, and may reflect a nonspecific peri-procedural response rather than infection. Depressive symptoms were recorded only in the control group and were clinically interpreted as being related to persistent PHS, delayed mobilization, and prolonged hospitalization; however, causality cannot be inferred from these retrospective data. Therefore, PEBP appears not only to effectively prevent the target complication of cerebrospinal fluid leakage but also to mitigate several indirect adverse sequelae inherent to standard conservative care. It should be emphasized that the demonstrated superiority of PEBP over conservative management in this study does not imply its superiority over other potential prophylactic materials or techniques, highlighting the need for future head-to-head comparative trials.

Exploratory subgroup analyses showed directionally similar associations across tumor stage and albumin strata. Advanced TNM stage reflects greater disease burden, while hypoalbuminemia is a marker of malnutrition, systemic inflammation, and poorer outcomes in cancer patients (26, 27). These variables were clinically relevant matching and subgroup factors but should not be interpreted as direct evidence of impaired dural healing.

Several methodological features support the credibility of the observed associations. Propensity score matching improved balance in recorded baseline and procedural covariates (28). This study also addresses an understudied, high-risk population across multiple centers. The validity and generalizability of our findings are strengthened by the use of objective diagnostic criteria for CSF leakage (MRI myelography) (29), comprehensive outcome assessments, and rigorous subgroup and survival analyses. Outcome classification included blinded verification, and subgroup and survival analyses were presented as exploratory supportive analyses.

The limitations of this study must also be acknowledged. First, the retrospective, multi-center design leaves potential for residual confounding despite statistical adjustment. E-values contextualize sensitivity to unmeasured confounding but do not eliminate it. Intraoperative factors such as needle gauge, puncture attempts, visible CSF leakage, and operator experience were not recorded and could have influenced both PEBP use and CSF leakage risk. Thus, confounding by indication remains the central methodological vulnerability, and PSM cannot adjust for unrecorded variables. Second, the matched estimates apply to the common-support population rather than all enrolled controls because 54/132 controls were excluded by the matching caliper. Third, the median follow-up duration was limited to 3 months, which is adequate for capturing acute CSF leakage complications but insufficient to definitively rule out rare or delayed events such as neuraxial seeding. Therefore, safety claims regarding seeding are limited to “no clinically evident neuraxial seeding during short-term follow-up.” Fourth, MRI myelography was clinically triggered rather than protocol-driven, which may have introduced verification/ascertainment bias and undercounted imaging-confirmed leakage in the PEBP group. Fifth, adherence to conservative management could not be objectively verified. Sixth, standardized pain-intensity and quality-of-life outcomes at 1 and 3 months after discharge were not consistently collected, so sustained patient-centered benefit could not be reliably assessed. Finally, our study employed a standardized PEBP protocol (10–15 mL autologous blood); the optimal volume, timing, and potential need for protocol individualization remain important questions for future research. Accordingly, these findings should be interpreted as adjusted associations consistent with benefit rather than definitive evidence of superiority or causal proof.

Future prospective studies should include randomized allocation where feasible, standardized recording of intraoperative factors, protocol-driven imaging criteria, longitudinal patient-centered outcomes, and longer follow-up to assess delayed risks. Mechanistic investigations could elucidate the precise biological actions of autologous blood components in the oncological dural repair milieu. Additionally, health economic analyses would be valuable to assess the cost-effectiveness of implementing PEBP as a routine preventive strategy.

## 5 Conclusion

In patients with refractory cancer pain undergoing intrathecal drug delivery system implantation, intraoperative prophylactic epidural blood patch was associated with lower rates of early cerebrospinal fluid leakage-related complications, less subsequent therapeutic intervention, and shorter hospital stay after adjustment for recorded covariates. Exploratory subgroup findings were directionally consistent across advanced disease and lower albumin strata. These findings support further evaluation of PEBP as a potential preventive strategy in carefully selected patients. Prospective, multicenter randomized trials are warranted to confirm these results and guide the optimization of perioperative care for this vulnerable patient group.

## Data Availability

The original contributions presented in the study are included in the article/supplementary material, further inquiries can be directed to the corresponding author.

## Glossary

AE: Adverse Event
ARR: Absolute Risk Reduction
BMI: Body Mass Index
CI: Confidence Interval
CSF: Cerebrospinal Fluid
EBP: Epidural Blood Patch
GI: Gastrointestinal (tumors)
HES: Hydroxyethyl Starch
IDDS: Intrathecal Drug Delivery System
IL-6: Interleukin-6
IQR: Interquartile Range
LOS: Length of Stay
MD: Mean Difference
MRI: Magnetic Resonance Imaging
NNT: Number Needed to Treat
NRS: Numerical Rating Scale
PDGF: Platelet-Derived Growth Factor
PEBP: Prophylactic Epidural Blood Patch
PHS: Postural Headache Syndrome
PSM: Propensity Score Matching
RCP: Refractory Cancer Pain
RR: Relative Risk
SD: Standard Deviation
SMD: Standardized Mean Difference
TGF-β: Transforming Growth Factor-beta
TNF-α: Tumor Necrosis Factor-alpha
TNM: Tumor-Node-Metastasis (staging)
