# Supplementary material for: Prophylactic epidural blood patch for cerebrospinal fluid leakage after intrathecal drug delivery system implantation in patients with refractory cancer pain: a multi-center retrospective cohort study
Source: Front Neurol. 2026 Jul 16;17:1883156. doi: 10.3389/fneur.2026.1883156 (PMC13420729; doi:10.3389/fneur.2026.1883156)
Supplement: Supplementary file 1 [file Supplementary_file_1.docx]

**Supplementary materials**

**Supplementary Figure S1** and **Supplementary** **Tables S1–S3** inside the manuscript are as follows:


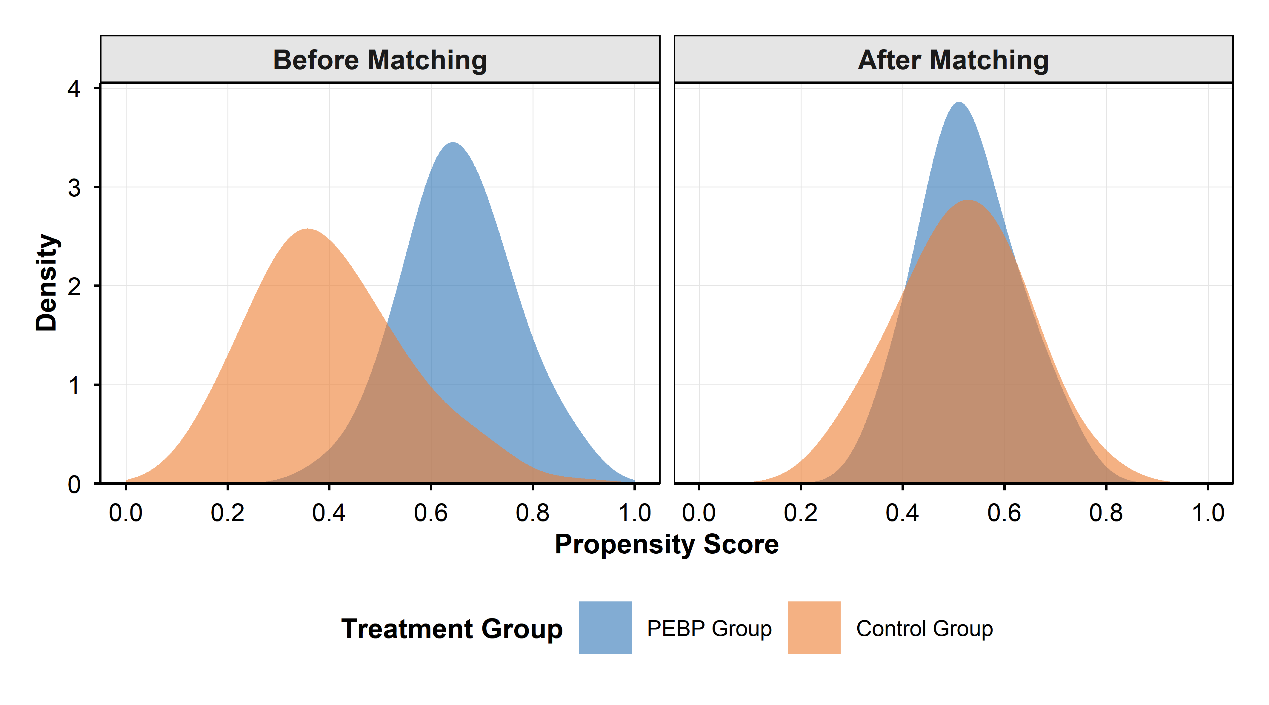


**Supplementary Figure S1.** Propensity score distribution before and after matching.

**Supplementary Table S1.** Distribution of included cases by center and year of diagnosis/treatment during the observation period (before matching)

| **Center** | **Year** | **PEBP (n)** | **Control (n)** | **Total (n)** |
| --- | --- | --- | --- | --- |
| Sichuan Cancer Hospital & Institute, Chengdu | 2023 | 12 | 13 | 25 |
|  | 2024 | 15 | 15 | 30 |
|  | 2025 | 13 | 12 | 25 |
| Sichuan Gem Flower Hospital, Chengdu | 2023 | 3 | 7 | 10 |
|  | 2024 | 4 | 8 | 12 |
|  | 2025 | 3 | 5 | 8 |
| Chengdu Xinhua Hospital, Chengdu | 2023 | 2 | 7 | 9 |
|  | 2024 | 3 | 7 | 10 |
|  | 2025 | 3 | 6 | 9 |
| The Third Affiliated Hospital of Guangxi University of Chinese Medicine, Liuzhou | 2023 | 4 | 4 | 8 |
|  | 2024 | 4 | 5 | 9 |
|  | 2025 | 4 | 4 | 8 |
| The People's Hospital of Leshan, Leshan | 2023 | 3 | 7 | 10 |
|  | 2024 | 4 | 6 | 10 |
|  | 2025 | 3 | 7 | 10 |
| Yanjiang District People's Hospital, Ziyang | 2023 | 2 | 6 | 8 |
|  | 2024 | 2 | 7 | 9 |
|  | 2025 | 2 | 6 | 8 |
| **Total 2023** | | 26 | 44 | 70 |
| **Total 2024** | | 32 | 48 | 80 |
| **Total 2025** | | 28 | 40 | 68 |
| **All years** | | 86 | 132 | 218 |

*PEBP, prophylactic epidural blood patch.*

**Supplementary** **Table S2.** Baseline Characteristics of Included vs. Excluded Patients

| **Characteristic** | **Included Patients (n=218)** | **Excluded Patients (n=78)** | ***P*-Value** | **SMD** |
| --- | --- | --- | --- | --- |
| Age, years (mean ± SD) | 60.1 ± 9.0 | 59.8 ± 8.7 | 0.801 | 0.03 |
| Gender, n (%) |  |  | 0.934 | 0.02 |
| Male | 125 (57.3) | 45 (57.7) |  |  |
| Female | 93 (42.7) | 33 (42.3) |  |  |
| BMI, kg/m² (mean ± SD) | 21.8 ± 3.4 | 21.5 ± 3.2 | 0.512 | 0.09 |
| Preoperative albumin, g/L (mean ± SD) | 34.2 ± 5.0 | 33.9 ± 4.8 | 0.675 | 0.06 |
| TNM stage, n (%) |  |  | 0.623 | 0.08 |
| Stage III | 68 (31.2) | 25 (32.1) |  |  |
| Stage IV | 150 (68.8) | 53 (67.9) |  |  |
| Spinal metastasis, n (%) | 0 (0) | 12 (15.4) | < 0.001 | 0.65 |
| Coagulopathy, n (%) | 0 (0) | 10 (12.8) | < 0.001 | 0.58 |
| Active infection, n (%) | 0 (0) | 8 (10.3) | < 0.001 | 0.52 |
| Incomplete data/loss to follow-up, n (%) | 0 (0) | 48 (61.5) | < 0.001 | 1.85 |

*Data presented as mean ± SD, or n (%). SMD， Standardized Mean Difference. P values from t-tests for continuous variables and χ² tests for categorical variables.*

**Supplementary Table S3.** E-values for the primary outcome of moderate-to-severe PHS

| **Outcome** | **Effect estimate (RR, 95% CI)** | **E-value for point estimate** | **E-value for 95% CI limit*** |
| --- | --- | --- | --- |
| Moderate-to-severe PHS | 0.25 (0.10–0.63) | 7.46 | 2.55 |

**The E-value for the confidence interval limit is closest to the null (upper bound of 95% CI). An unmeasured confounder associated with both treatment and outcome by risk ratios of 2.55 each could shift the confidence interval to include the null, but a stronger confounder (7.46) would be needed to explain away the point estimate. Abbreviations: RR, relative risk; CI, confidence interval; PHS, postural headache syndrome.*
